# Determining the Impact of a School-Based Health Education Package for Prevention of Intestinal Worm Infections in the Philippines: Protocol for a Cluster Randomized Intervention Trial

**DOI:** 10.2196/18419

**Published:** 2020-06-25

**Authors:** Mary Lorraine S Mationg, Gail M Williams, Veronica L Tallo, Remigio M Olveda, Eindra Aung, Portia Alday, Mark Donald Reñosa, Chona Mae Daga, Jhoys Landicho, Maria Paz Demonteverde, Eunice Dianne Santos, Thea Andrea Bravo, Franziska A Angly Bieri, Yuesheng Li, Archie C A Clements, Peter Steinmann, Kate Halton, Donald E Stewart, Donald P McManus, Darren J Gray

**Affiliations:** 1 Research School of Population Heath The Australian National University Canberra Australia; 2 Department of Epidemiology and Biostatistics Research Institute for Tropical Medicine Manila Philippines; 3 School of Public Health University of Queensland Brisbane Australia; 4 St Vincent’s Clinical School University of New South Wales Sydney Australia; 5 Molecular Parasitology Laboratory Infectious Diseases Division QIMR Berghofer Medical Research Institute Brisbane Australia; 6 Hunan Institute of Parasitic Diseases World Health Organization Collaborating Centre for Research and Control on Schistosomiasis in Lake Region Yueyang China; 7 Faculty of Health Sciences Curtin University Perth Australia; 8 Swiss Tropical and Public Health Institute Basel Switzerland; 9 University of Basel Basel Switzerland; 10 School of Public Health and Social Work Queensland University of Technology Brisbane Australia; 11 School of Medicine Griffith University Brisbane Australia

**Keywords:** soil-transmitted helminths, school-based health educational intervention, Magic Glasses, integrated control, randomized controlled trial, Philippines

## Abstract

**Background:**

Repeated mass drug administration (MDA) of antihelminthics to at-risk populations is still the main strategy for the control of soil-transmitted helminth (STH) infections. However, MDA, as a stand-alone intervention, does not prevent reinfection. Accordingly, complementary measures to prevent STH reinfection, such as health education and improved sanitation, as part of an integrated control approach, are required to augment the effectiveness of MDA for optimal efficiency and sustainability.

**Objective:**

The aim of this study is to determine the impact and generalizability of a school-based health education package entitled *The Magic Glasses* for STH prevention in the Philippines.

**Methods:**

We conducted a cluster randomized controlled intervention trial, involving 2020 schoolchildren aged 9-10 years, in 40 schools in Laguna Province, Philippines, to evaluate the impact of the school-based health education package for the prevention of STHs. The trial was conducted over the course of 1 year (June 2016 to July 2017). A total of 20 schools were randomly assigned to the intervention arm, in which *The Magic Glasses Philippines* health education package was delivered with the standard health education activities endorsed by the Philippines Department of Health (DOH) and the Department of Education (DepEd). The other 20 schools comprised the control arm of the study, where the DOH/DepEd’s standard health education activities were done. At baseline, parasitological assessments and a knowledge, attitude, and practice survey were carried out in all schools. In addition, height, weight, and hemoglobin levels were obtained from each child (after parental consent), and their school attendance and academic performance in English and mathematics were accessed from the school records. The baseline and 2 follow-up surveys were completed using the same study measurements and quality-control assessments.

**Results:**

Key results from this cluster randomized intervention trial will shed light on the impact that *The Magic Glasses* health education package will have against STH infections in schoolchildren in the province of Laguna, located on the Island of Luzon, in the Calabarzon Region of the Philippines.

**Conclusions:**

The results of the trial will be used to assess the generalizability of the impact of *The Magic Glasses* health education package in different epidemiological and cultural settings, providing evidence for translation of this health education package into public health policy and practice in the Asian region and beyond.

**Trial Registration:**

Australian New Zealand Clinical Trials Registry number ACTRN12616000508471; https://www.anzctr.org.au/Trial/Registration/TrialReview.aspx?id=368849

**International Registered Report Identifier (IRRID):**

DERR1-10.2196/18419

## Introduction

Soil-transmitted helminth (STH) infections, including roundworms (*Ascaris lumbricoides*), whipworms (*Trichuris trichiura*), and hookworm (*Necator americanus* and *Ancylostoma duodenale*), affect more than a quarter of the world’s population, particularly inhabitants of poorer regions [1]. The highest STH prevalence occurs in Central and South America, the People’s Republic of China, Southeast Asia, and sub-Saharan Africa [1]. STH infections are commonly associated with poverty, where access to satisfactory sanitation, adequate waste disposal, clean water, hygiene, and health care is poor and knowledge of preventive measures through health education is inadequate [2-5]. The public health importance of STH infections is widely recognized, as they are associated with malnutrition, poor growth and development, iron-deﬁciency anemia, diminished physical ﬁtness, and impaired cognitive development [3-7]. These features are of particular concern in schoolchildren, who have the highest infection prevalence and intensity of *A. lumbricoides* and *T. trichiura* and are at risk of a high burden of hookworm-associated morbidity [3,5,8]. The most recent estimate (2015) of disability-adjusted life years lost to STH infections is about 3.38 million years worldwide [9].

The World Health Organization (WHO) recommends preventive chemotherapy as a means to control STH infections. This strategy is implemented through mass drug administration (MDA) with benzimidazoles, compounds which are cheap and safe but have variable efficacy depending on the STH species and location. The main feature of this strategy is to administer MDA regularly to at-risk populations with a target of treating at least 75% of preschool-aged children (pre-SAC) and school-aged children (SAC). The WHO recommends annual MDA of pre-SAC and SAC in areas where the prevalence of STH is between 20% and 50% and semiannual if above 50% are infected [10]. The London Declaration on Neglected Tropical Diseases (NTDs) included a pledge from pharmaceutical companies to continue their donations of antihelminthic drugs until 2020 [11,12]. With this commitment, the worldwide deworming coverage has greatly increased over the past years, but the deworming coverage target of 75% has yet to be reached [12]. As a stand-alone intervention, the MDA strategy decreases the intensity and severity of infection and improves the health and nutrient uptake of children [1,10], but it does not prevent reinfection [13-15]. Repetitive treatment is required as the eggs or larvae of intestinal worms continuously contaminate the external environment for many months, and poor hygiene and sanitation favor recurrent exposure [16]. Although WHO advocates repeated rounds of chemotherapy in areas where MDA programs have stopped, infection prevalence and intensity have rapidly rebounded to pretreatment levels [14,17-19]. This lack of sustained benefit substantially lessens the effectiveness of MDA.

Recent mathematical modeling studies of STH transmission show that MDA programs targeting pre-SAC and SAC alone cannot eliminate STH infections; adults must also be treated at high coverage levels [13,20,21]. Other studies have shown that in areas with high STH transmission, high coverage and frequency of treatment [22], with health education and water, sanitation, and hygiene (WASH) efforts are required [14]. To sustain MDA as a stand-alone strategy, an uninterrupted supply of antihelminthic drugs is essential. Whether large-scale drug donations will continue beyond 2020 remains unclear [12], but without continuous donor support, MDA may not be sustainable in the long run. Another concern with MDA includes the potential development of drug resistance as a result of continued treatment pressure on the parasites [14,23]; indeed, there have been reports of decreased drug efficacy against hookworms [24-26] and *T. trichiura* [17,27,28]. With the continual threat of drug resistance, developing complementary interventions for preventing STH reinfection, such as improvements in personal hygiene through health education, as part of an integrated approach, are required to complement chemotherapy to treat and prevent STH infections. This will reduce the number of treatment rounds necessary, consequently lessening the treatment burden and thus creating a more sustainable long-standing approach to STH control.

In 2013, we reported the successful development and testing of a health education package featuring a 12-minute animated narrative cartoon video entitled *The Magic Glasses* to prevent STH infections in Chinese primary school children [29]. The cluster randomized controlled intervention trial, conducted in Linxiang City District, Hunan Province, People’s Republic of China, and involving 1718 children in 38 rural schools showed that the video-based health education package increased students’ knowledge about STH and led to behavior change and a 50% efficacy in preventing STH infections [29]. To evaluate the potential for up-scaling this video-based health educational package as a universal school-focused educational tool to form part of a multicomponent sustainable STH control program, we assessed the generalizability of the earlier findings in different geographical settings with a greater force of infection and in different sociocultural groups. This is to provide an evidence base for the translation of the package into public health policy and practice in the Asian region and beyond.

In June 2016, we commenced a new trial to assess the impact of the video-based educational package, culturally adapted for the Philippines. This report describes the protocol of *The Magic Glasses Philippines* (MGP) trial, which has been developed using the Standard Protocol Items: Recommendations for Intervention Trials 2013 guidelines (see Multimedia Appendix 1) [30].

## Methods

### Study Design

**Figure 1:**
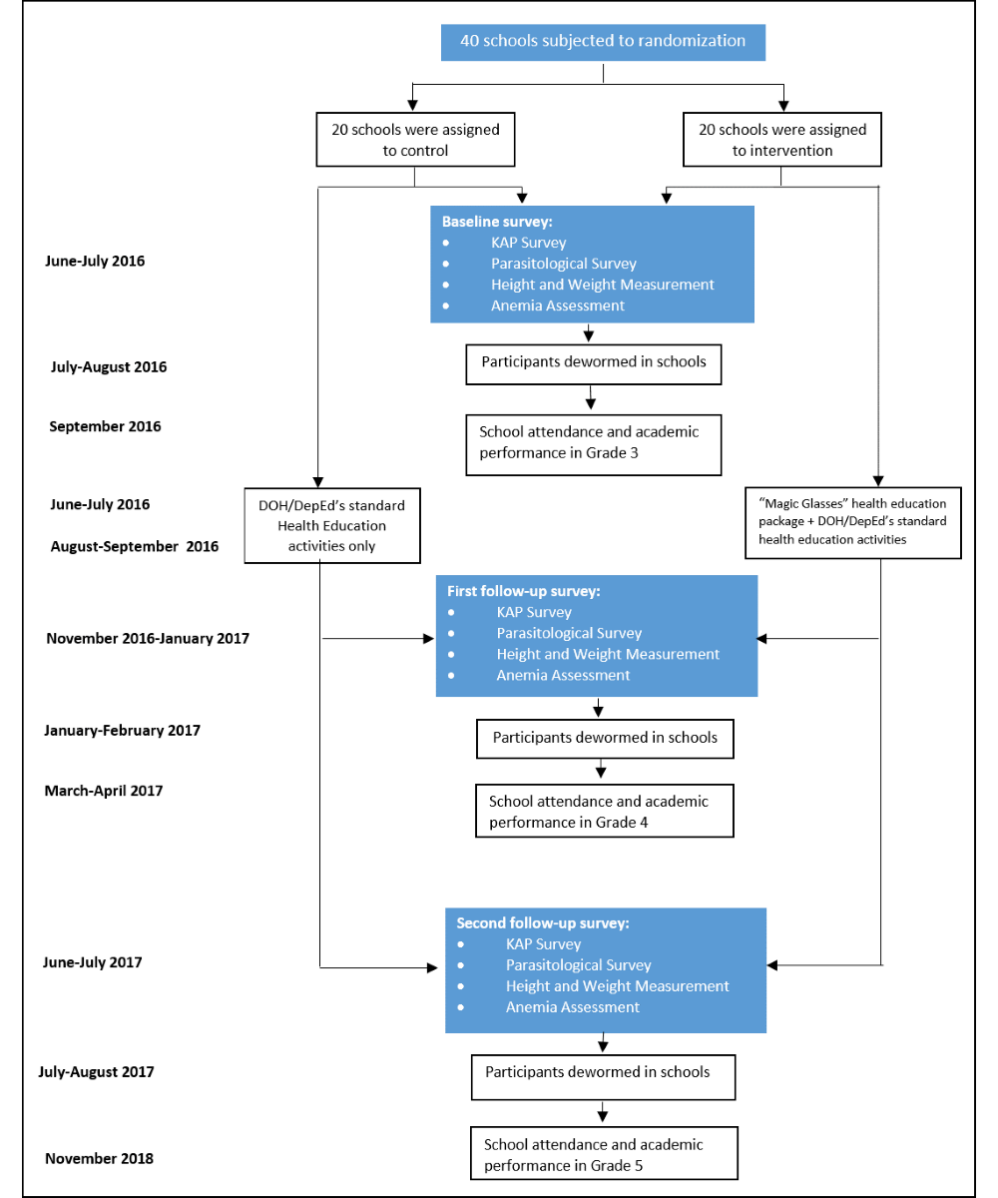
Trial study design and timelines. DepED: Department of Education; DOH: Department of Health; KAP: knowledge, attitude, and practice.

Our overarching hypothesis is that a video-based health educational package (for use in schools) targeting STH will increase students’ knowledge of intestinal worms, their transmission, symptoms, treatment, and prevention and improve self-reported hygiene behavior. We addressed this hypothesis by conducting an unmatched cluster randomized intervention trial, targeting schoolchildren aged 9 to 10 years, in 40 schools in Laguna province, the Philippines. The trial study design is shown in Figure 1. The trial was conducted over the course of 1 year (June 2016 to July 2017). A total of 20 schools were randomly assigned to the *intervention* arm, in which the *MGP* health education package was delivered in combination with the standard health education activities (focused on WASH) endorsed by the Philippines Department of Health (DOH) and the Department of Education (DepEd). The other 20 schools comprised the *control* arm of the study, where the DOH/DepEd’s standard health education activities were implemented. At baseline, parasitological assessments and a STH-related knowledge, attitude, and practice (KAP) survey were carried out in all schools. In addition, height, weight, and hemoglobin levels were obtained from each consenting child; school attendance and academic performance were accessed from the school records. Only those children who had matched data on KAP and at least one stool sample at baseline were deemed eligible for assessments in the 2 follow-up surveys. The baseline (June-July 2016), first follow-up (Nov 2016-Jan 2017), and second follow-up (June-July 2017) surveys were completed using the same study measurements and quality-control assessments. The baseline survey was conducted immediately before the semiannual mass administration of albendazole in all schools, and follow-up surveys were conducted 5 months before the MDA [31].

### Study Setting

Approximately 43 million children are at risk of STH infection in the Philippines (2016 figures, calculated using the Preventive Chemotherapy Databank, maintained by the WHO); of these, about 24% are pre-SAC, whereas 76% are SAC [32]. The burden caused by STH infections remains high according to the results of several studies conducted in the country. A nationwide survey performed in 2001 and reported in 2005 found that the STH prevalence among schoolchildren was as high as 67% [33]. A subsequent study in 2006 showed a prevalence of 54% for at least one type of STH infection and a prevalence of 23.1% for heavy-intensity infections [34]. In a follow-up survey in 2009, a decrease in the overall prevalence (44.7%) and heavy-intensity STH infections (19.7%) among SAC (aged 6-12 years) was reported [35]. Although the prevalence had decreased, it still exceeded the 20% target for morbidity control set forth by the WHO.

The current Philippines national control program for STHs involves semiannual MDA for schoolchildren aged 6-12 years in all public elementary schools (ESs), as advocated by the WHO. This has been implemented since 2007 by the DepEd in collaboration with the DOH through its Integrated Helminth Control Program (IHCP) [36]. As part of the long-term strategy, the IHCP also incorporates health education/promotion and WASH interventions through the DepEd [37,38]. However, the persistently high prevalence of STH reported across the country suggests that the impact was lower than expected [39,40]. In 2014, we conducted a cross-sectional pilot survey in the province of Laguna (located on the Island of Luzon, in the Calabarzon Region of the Philippines). The survey was undertaken to confirm the appropriateness of the study site for the MGP trial by quantifying the prevalence of STH among ES children, particularly before the first implementation of the National School Deworming Day program on July 29, 2015 [41]. The results of the survey showed a prevalence of 33.3% by the Kato-Katz (KK) thick fecal smear microscopy technique for at least one type of STH infection [40]. Overall, the pilot survey indicated that Laguna satisfied the criteria as a study site to test the MGP and that STH continues to be a significant public health concern in the Philippines despite several years of MDA implementation.

### Ethics Approval and Consent to Participate

The study protocol was submitted to and approved by the institutional review board of the Research Institute for Tropical Medicine (RITM) with approval number 2013–16, the QIMR Berghofer Medical Research Institute (QIMRB) Human Ethics Committee (approval number: P1271), and the Australian National University Human Ethics Committee (approval number: 2014/356). Permission was sought from the DepEd and DOH before the conduct of the study. With the permission obtained, we provided an orientation about the study to the principals of each school involved, and their oral consent was sought to participate in the study. Written informed consent was obtained from the parents or legal guardians of the students invited to participate in the study. The purpose and procedures of the study were also explained to the participating children, and their oral assent was sought.

### Intervention Program: Magic Glasses Philippines

The existing educational video *The Magic Glasses*, originally for Chinese schoolchildren [42], was culturally adapted for the Philippines setting. The cultural adaptation of the video involved 3 major steps: formative research, production and pilot testing, and revision.

#### Formative Research

The formative research was carried out in selected schools (outside the main trial study area) in 3 municipalities of Los Baños, Pagsanjan, and Victoria in the province of Laguna from August to September 2014. It included an initial assessment of the previous KAP of the schoolchildren using a quantitative questionnaire, qualitative drawing assessment, field observations, and interviews to identify risk factors and drivers for behavior change to translate them into key messages for the video. The formative research comprised the following data collection procedures:

1. A household survey was conducted involving 30 households with Grade 4 children from 3 randomly selected municipalities mentioned above (involving 10 households in a selected village per municipality). The survey involved household observations, in-depth interviews with the head of the household, and infrastructure assessments.
2. A KAP questionnaire was administered to Grade 4 and Grade 5 schoolchildren (aged 9-10 years) in 10 randomly selected schools in the same 3 municipalities (N=616) to assess their KAP associated with intestinal worm infections. Information on the cartoon preferences of schoolchildren was also collected in this survey.
3. Qualitative *draw and write* assessment and semistructured interviews with 30 schoolchildren in 3 selected schools in the same municipalities (10 schoolchildren per school) were conducted to assess their previous knowledge on intestinal worms.
4. Key informant interviews were conducted with teachers (n=6; 1 district supervisor, 2 principals, 2 language teachers, and 1 mathematics teacher), doctors (n=2; 1 municipal health officer and 1 pediatrician), and nurses (n=2; 2 health education promotion officers).

#### Production of the Video

The cartoon video was developed from October 2014 to May 2015. First, a review of the history of Philippines animation and popular Philippines cartoons was conducted. This information and the results from the formative research were then used to adapt the script and storyboard from the original video to the Philippines culture. Behavioral theories such as the Health Belief Model [43], Integrated Behavioral Model [43,44], and Social Cognitive Theory [43,44] were explored to ensure that the final cartoon was both engaging and informative. Following the script and storyboard adaption, the concept art was developed, and the animation process was finalized using Adobe Creative Suite (Adobe Systems Incorporated), Autodesk 3DS Max (Autodesk, Inc), and Motion-builder software (Autodesk, Inc). The audio for the video was recorded and dubbed by Filipino university students from the University of the Philippines Los Baños.

#### Pilot Testing of the Video

In 2015, the beta version of *The Magic Glasses Philippines* video was piloted in 2 schools in Laguna Province, again located outside the main trial area: one rural area (San Isidro ES in Calauan) and one urban area (Sampaloc ES in Pagsanjan). The cartoon was shown twice to an audience of schoolchildren (n=124) and teachers (n=7) in the 2 schools. The schoolchildren completed a short questionnaire during the second viewing session to assess whether the key messages of the video were understood. Subsequently, a classroom discussion was conducted to take note of any existing questions and comments. A total of 10 randomly selected schoolchildren in each school (n=20) were also invited to a focus group discussion (FGD) to identify comprehension problems and to seek feedback in a smaller group. Teachers from both schools (n=3 for San Isidro ES and n=4 for Sampaloc ES) were also asked to answer questions related to the content of the video, visual and audio aspects, and its cultural acceptability. The answers in the questionnaire were discussed in the FGDs for both groups (schoolchildren and teachers), and participants were asked to comment on the cartoons and make suggestions for its improvement.

To ensure that the cartoon had been appropriately adapted and technical issues had been rectified, the revised version of the cartoon was piloted again in 2 schools (outside the radius of 3 km from the main trial sites): one in a rural municipality (Dayap ES in Calauan) and the other in an urban municipality (Platero ES in Biñan). The revised version of the video was shown to an audience of 113 schoolchildren, 18 parents, and 4 teachers in the 2 schools. FGDs were conducted with 10 schoolchildren in each school (n=20) and parents (n=8 for Dayap ES and n=10 for Platero ES), whereas individual interviews were conducted with the teachers in each school (n=2 for Dayap ES and n=2 for Platero ES).

#### Delivery of the Intervention Program (Within the Trial)

**Figure 2:**
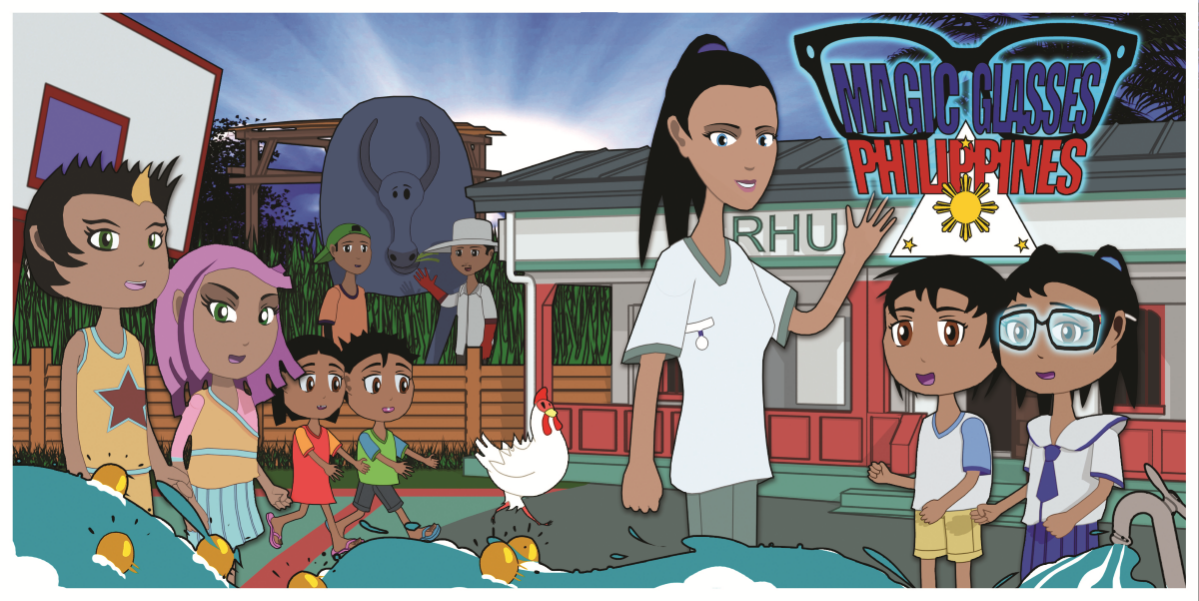
Cover of the cartoon “The Magic Glasses Philippines”.

The intervention schools received the video-based health educational package, which was administered by the research staff. The presentation of the cartoon was supplemented by a classroom discussion, a pamphlet summarizing the key messages delivered in the cartoon, and a drawing and essay-writing competition to reinforce the messages. Details of the implementation of the health education package are shown in Table 1. The front cover of the cartoon *The Magic Glasses Philippines* is shown in Figure 2.

**Table 1:** Details of the implementation of the health education package for soil-transmitted helminths in the intervention schools.

Date and education component		Aim
**May 2016**		
	Research staff training	The research staff were oriented on how to deliver the health education package
**June 2016**		
	Baseline survey	N/A^a^
**June-July 2016**		
	Video shown twice	Inform about STH^b^ transmission and prevention
	Student questions	Repeat key messages and answer students’ questions
	Distribution of pamphlet (comic)	Key messages as take-home message
**July-August 2016**		
	Participants received treatment at school (as part of the National Deworming Month program)	N/A
**August-September 2016**		
	Video shown twice	Reinforce knowledge about STH transmission and prevention
	Student questions 10-15 min classroom discussion based on student questions	Repeat key messages and answer students’ questions
**August-September 2016**		
	Essay competition; write story about own actions taken to prevent worm infection	Practice and reinforce new knowledge
**November 2016-January 2017**		
	First follow-up survey	N/A
**January-February 2017**		
	Participants received treatment at school (as part of the National Deworming Month program)	N/A
**June-July 2017**		
	Second follow-up survey	N/A
**July-August 2017**		
	Participants received treatment at school (as part of the National Deworming Month program)	N/A

^a^Not applicable.

^b^STH: soil-transmitted helminth.

#### Standard Intervention Approach

Both the intervention and the control schools received the standard health education activities as part of the WASH in Schools (WINS) Program for the promotion of correct hygiene and sanitation practices among schoolchildren endorsed by DepEd and DOH. This program covers the following provisions in schools: (1) provision of safe water, hand washing, toilet, and proper drainage facilities; (2) proper hand washing; (3) oral hygiene; (4) food sanitation; (5) deworming; (6) environmental sanitation; (7) menstrual hygiene management; (8) solid waste management; (9) capacity building for program implementation; and (10) health education focused on hygiene and sanitation. The key concepts of the WINS program are incorporated into the kindergarten to Grade 12 curriculum (ie, in the case of the study participants included in the trial, it was integrated under the Health Education subject in Grade 4) [45]. Teachings included in the Health Education subject are correct knowledge and understanding of the importance of proper hygiene and sanitation practices.

#### Mass Drug Administration

Following the baseline survey, the recruited students across both control and intervention schools were treated with the WHO recommended dose (400 mg) of albendazole as part of the National Deworming Month program. In the intervention schools, MDA occurred simultaneously with the delivery of *The Magic Glasses* intervention. Follow-up surveys were scheduled to occur just before the semiannual National Deworming Month program.

### Study Outcomes

The primary outcomes of the study are STH infections rates and knowledge of intestinal worms and their transmission, symptoms, treatment. Secondary outcomes are changes in self-reported behavior (hand washing, use of toilets, and food hygiene). Tertiary outcomes comprised measures of morbidity (hemoglobin levels to assess anemia; height and weight for stunted growth and malnutrition) and school academic performance and attendance for impact on education. All outcomes are compared between the control and intervention schools.

### Selection of Clusters/Schools

Sample size calculations were performed according to the study by Hayes and Bennett [46]. With an infection incidence of 18% and an intervention efficacy of 30%, the study had 80% power with a sample size of 20 intervention clusters (40 in total) and 38 students per cluster at the end of the trial, with a predicted annual 10% loss to follow-up.

**Figure 3:**
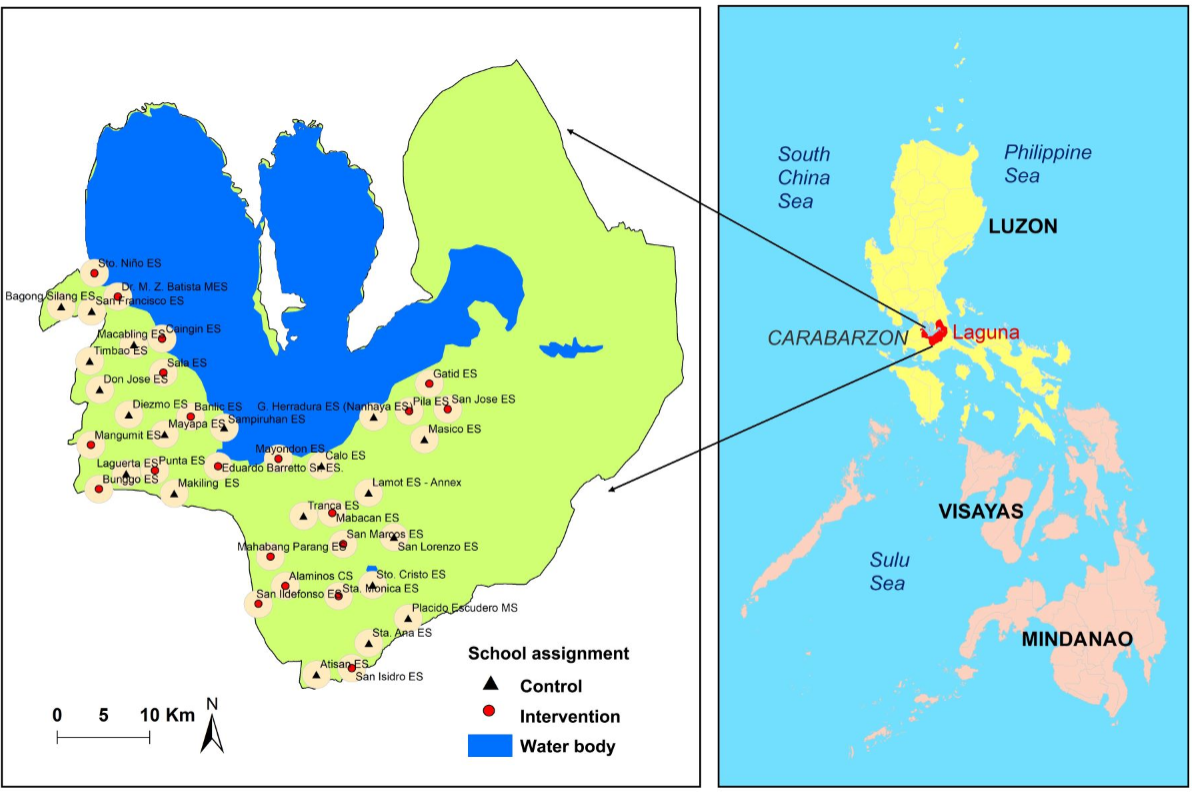
Study site and school locations. Map generated through ArcGIS program (ESRI Inc, Redlands, CA, USA).

Participating public ESs were selected using a spatial sampling technique to eliminate contamination between the intervention and control schools. From the list of schools that fit the criteria of 3 km radius distance from each other, 40 schools were randomly selected and assigned either to the intervention (MGP health education package) group or to the control group (no MGP health education package). Thus, 20 schools were randomly assigned to the intervention group, leaving the remaining 20 as controls. Figure 3 shows a map of the school locations.

### Recruitment of Study Participants

Study participants were Grade 4 students (aged 9-10 years) from the selected schools. To ensure that all parents were engaged and informed about the study, the research team, with the assistance of the village or barangay health workers (BHWs), organized parents and caregivers’ meetings at the catchment barangays of the schools included in the MGP trial before the commencement of classes in June 2016. The BHWs who assisted in the parents/caregiver’s meeting were oriented by the research staff on STH and the MGP study. The STH orientation aimed to empower the BHWs about intestinal worms and relate these with preventive measures in other DOH programs, such as sanitation and hygiene. The orientation about the MGP study provided the BHWs with the information necessary to invite and ask the parents or caregivers to attend the meetings. The research staff were responsible for obtaining parental informed consent after the parents and caregivers’ meeting. Caregivers who were not reached through the caregiver’s meetings were located and oriented by the BHWs in their homes; the research staff validated the information provided to these parents and caregivers during home visits and obtained written consent.

One week before the baseline survey, the Grade 4 students in both the intervention and control schools were oriented about the study and the different study procedures to be conducted on them. However, information regarding cartoon viewing was provided only in the intervention schools. An informed consent document was also distributed to the students whose parents were not present during the parents and caregivers’ meeting in the barangay. These students were asked to return the signed informed consent form to the research staff on the day of the survey. The inclusion criteria for schoolchildren were as follows: (1) enrolled in Grade 4 and (2) had parental informed consent.

### Data Collection

#### Field Organization, Training, and Field Schedule

Four teams were set up and organized to carry out the data collection procedures in the 40 participating schools before the commencement of the National Deworming Month. Each team, composed of 1 supervisor from the Department of Epidemiology and Biostatistics from the RITM in the Philippines, 1 team leader, and 6 medical technologists, was responsible for collecting data from 2 to 3 schools per week.

Before the start of the survey, the research staff had undergone a 2-week training. The first week was a microscopy proficiency training conducted by the National Reference Laboratory for Parasitology of RITM, whereas the second week focused on data collection procedures, filling out of forms, and administration of the data collection tools.

Four visits were made at each school at baseline and during the 2 follow-up surveys to allow the children with multiple opportunities to participate in all the data collection procedures, if they were absent during previous visits. On the fifth day, a review of the data collection forms was done to ensure accuracy, consistency, and completeness of the information collected before the departure of each team from the schools and moving to the next set of schools.

#### Stool Surveys

Stool samples were collected at baseline and at 2 follow-up surveys. A week before the survey week, the participating children, following their orientation to the study, were requested to provide 2 stool samples as part of their involvement in the project. Each child was given a stool collection kit that included a stool container, gloves, an applicator stick, and instructions on how to collect a stool sample. Children were instructed to collect 2 stool samples on any morning of the 4 collection days. They were asked to submit only 1 sample per day (normally in the morning of the same day of collection) to the research staff in the school. The samples were collected, processed at the school site within 2 hours after collection, and read the same day using 3 KK thick smears (41.7 mg of stool/smear) prepared per sample [47]. A team of trained microscopists read the slides. The microscopists worked independently of each other on the samples assigned to them, with 1 sample examined by 1 microscopist only. The number of STH eggs was counted and recorded separately for each helminth species. To ensure the validity and accuracy of the results, 10% of all slides were randomly selected and reexamined by a reference microscopist on each collection day.

#### Knowledge, Attitude, and Practices Survey

All study participants were administered a questionnaire on STH-related KAP at baseline and at the 2 follow-up surveys. The KAP questionnaire consisted of multiple-choice and open-ended questions regarding demographics, health characteristics, medical history, and previous health education; knowledge about intestinal worms, how they are transmitted, and the symptoms and treatment of STH infection; the student’s attitude toward STH; self-reported hygiene practices with respect to hand washing, handling of food, using the toilet, and wearing of shoes; and household characteristics relating to household water source and household assets. The questionnaire was translated into Tagalog and back-translated to English to ensure accuracy. It was piloted in March 2015 in 2 schools outside the main trial area on a total of 83 schoolchildren from San Andres ES in the municipality of Alaminos (n=26) and Gulod ES in the municipality of Calauan (n=57).

Two research staff members were responsible for administering the questionnaire. One gave instructions to the children and read the questions one-by-one in front of the class, whereas the other moved around the room to check whether the children were able to follow the instructions and to ensure that each question was answered.

Students were considered to have a positive (or correct) attitude toward STH if they were aware of the risk of infection and intended to change their behavior to prevent an infection. Students were considered to have a negative (wrong) attitude if they did not recognize the health risk of STH and the importance of correct behavior (eg, good hygiene). A higher score in the questionnaire was considered indicative of a more positive attitude.

#### Anthropometric and Hemoglobin Measurements

Using a height scale chart (paper beam chart) and calibrated digital weighing scale (Tanita HD-383, Tanita Corporation, Japan), all participating children underwent measurement of height (to nearest 0.1 cm) and weight (to nearest 0.1 kg), respectively, obtained as a single measurement at baseline and at the 2 follow-up surveys. Fingerprick blood samples for hemoglobin measurement (using a portable hemoglobin analyzer [HemoCue Hb 301 System, HemoCue Sweden]) were also collected from 2000 randomly selected children with matching data on the KAP and at least one stool sample at baseline and at the 2 follow-up surveys. Using the height and weight measurements collected from the children, anthropometric values indicative of their nutritional status were calculated. Indicators for malnutrition included stunting (height-for-age), thinness (BMI-for-age), and being underweight (weight-for-age). These anthropometric indicators were calculated as Z scores (the number of SDs from the mean of the standard population, with malnutrition and severe malnutrition defined as values 2 and 3 SDs, respectively, below the mean score of the standard population [48,49]), employing the 2007 WHO growth standards for SAC and adolescents [49]. Anemia was defined according to the WHO classification guidelines, adjusted for altitude in communities more than 1000 m above sea level [50].

#### Collection of Attendance and Academic Performance

The attendance for the school year and academic performance based on grades or end-of-quarter marks in English and mathematics for each of the 4 grading periods of the participating children while they were in Grades 3, 4, and 5 were accessed from the school records and were obtained in September 2016, March 2017, and November 2018, respectively. The end-of-school-term marks were defined as the arithmetic mean of the 4 grading periods in each school year. The attendance rate was defined as the number of days the children had attended school over the total number of days in 1 school year.

#### Treatment

Upon completion of each survey (ie, baseline and first and second follow-up), parasitological and hemoglobin results were communicated to all parents in an enclosed envelope, with a recommendation for treatment (if necessary) at the local health center or a request to participate in the deworming activity at school. All participating children were encouraged to take the deworming drug (albendazole [400 mg], as recommended by the WHO) provided free of charge. The school principal, teachers, and/or assisting health professionals (nurses) at each school monitored the students for treatment compliance. In collaboration with the school principals and teachers, the research staff collected the data on the deworming status of the participating students, any side effects experienced, and the reason why any student failed to take the drug.

#### School Facility Survey

In 2015, before the main trial, a school facility survey was conducted, whereby Grade 4 classrooms in all 40 schools included in the MGP trial were visited and assessed for the presence of toilets, water facilities, and hand washing area, and their functionality.

#### Household Survey

A household survey was conducted by trained interviewers on a subset of randomly selected students (n=400; households of 10 schoolchildren from each of the 40 participating schools) between October 23 and November 22, 2017, to assess the household support and facilities that promoted STH preventive behavior among the participating schoolchildren. Informed consent was obtained from each student’s parent or caregiver, and a structured interview was administered to them. The structured interview covered questions related to knowledge on STH and health education, household assets, and household infrastructure related to WASH. Rapid assessments of the hand-washing facilities and toilets available in the selected households were also conducted.

### Data Quality Assurance and Processing

#### Quality Assurance of the Field Data Collection

Field data quality was monitored through quality assurance by research investigators. All questionnaires and data collection forms were reviewed for accuracy, consistency, and completeness. This review was undertaken immediately after data collection, before the respective research teams had left the area.

#### Management and Processing of Qualitative Data

All qualitative FGDs and interviews were conducted in English and Tagalog. Informed consent was obtained from all study participants (teachers and from the parents of children involved). All FGDs and interviews were audio recorded, transcribed verbatim, coded, and analyzed.

### Data Management and Confidentiality

All results collected from the study respondents were kept confidential. Stool samples were labeled using each participant’s assigned study ID number, with no identifying information. Reports that had been generated from this study only contained a summary of the data collection without the names of the respondents.

Results of parasitological assessments, KAP surveys, school attendance information, academic performance, deworming data, height, weight, and hemoglobin measurements were entered twice by 2 different data encoders in a customized password-protected data entry system developed using Microsoft Access [51]. The data entry system contained validation codes and built-in range checks for appropriate variables. The final study datasets were accessible only to the study investigators. All study forms were placed in a locked cabinet in a study office at RITM.

### Study End Point Analyses

Models for infection will use generalized estimating equations (GEEs), and a logistic regression model will be used to estimate odds ratio and, therefore, intervention efficacy against infection, accounting for clustering within schools, repeated measures, and baseline infection. Stratification by school-based baseline prevalence will be undertaken to evaluate intervention efficacy at low and high endemicity. Analyses of changes in knowledge and self-reported behavior scores will be analyzed with the use of a linear regression model and GEE to take into account clustering within schools and repeated measures. Potential confounders such as age, sex, and rural or urban status of schools will be incorporated. Spearman correlation coefficients will be used to estimate correlations among self-reported behavior, knowledge, and incidence.

### Dissemination

The progress and key results of the study were communicated to and discussed with the Philippines DOH, DepEd, other key decision makers and local stakeholders, including community members of the province of Laguna, at several workshops over the course of the trial.

## Results

The study enrollment was carried out in June 2016. Baseline, follow-up 1, and follow-up 2 surveys were completed between June and July 2016, November 2016 and Jan 2017, and June and July 2017, respectively. Data analysis is currently underway, and the first results are expected to be submitted for publication in 2020.

## Discussion

The current global context for the elimination of common NTDs is focused on chemotherapy-based control. With the limitations of stand-alone MDA approaches [24], the potential added benefits of including health education and WASH interventions in treatment programs for the prevention of STH infections (as part of an integrated multicomponent approach) for sustainable control must be emphasized. It has been repeatedly shown that to create an enabling environment for both chemotherapy and sanitation to thrive, additional public health measures, including novel, effective, simple, and low-cost health educational interventions, are needed [52-55].

The effective health education package *The Magic Glasses*, previously developed and successfully tested in schools in People’s Repblic (PR) of China [29], complements the current approach to control STH infections advocated by the WHO. The trial in PR China established proof of principle that the health education package increased the knowledge and changed the behavior of students, resulting in a significant decrease in their intestinal worm infections.

To demonstrate the generalizability of the approach, we culturally adapted *The Magic Glasses* video for the Philippines audience and applied the health education package in a single-blinded cluster randomized intervention trial to evaluate its impact on STH infection in schoolchildren in Laguna province. The goal was to establish proof of principle that the package is effective and applicable in a different geographical area with a greater prevalence of STH infection and in a different ethnic group. Furthermore, the sensitive cultural adaption of this tool provides an evidence base for a health educational package for use in schools that can be readily integrated into the school curriculum.

A more integrated comprehensive control strategy—combining MDA with improvements in hygiene through health education—has the potential to result in the sustainable control of STH infections among schoolchildren. This trial was designed to provide additional evidence supporting the inclusion of this health education package into public health policy and practice in the Asian region and beyond.

## Appendix

Multimedia Appendix 1 SPIRIT Checklist.

Multimedia Appendix 2 Existing Peer-Review Reports from Funding Agencies.

## Abbreviations

BHW: barangay health worker
DepEd: Department of Education
DOH: Department of Health
ES: elementary school
FGD: focus group discussion
GEE: generalized estimating equation
IHCP: Integrated Helminth Control Program
KAP: knowledge, attitude, and practice
KK: Kato-Katz
MDA: mass drug administration
MGP: Magic Glasses Philippines
NTD: neglected tropical disease
pre-SAC: preschool-aged children
RITM: Research Institute for Tropical Medicine
SAC: school-aged children
STH: soil-transmitted helminth
WASH: water, sanitation, and hygiene
WHO: World Health Organization
WINS: WASH in Schools
