# Supplementary material for: Determining the Impact of a School-Based Health Education Package for Prevention of Intestinal Worm Infections in the Philippines: Protocol for a Cluster Randomized Intervention Trial
Source: JMIR Res Protoc. 2020 Jun 25;9(6):e18419. doi: 10.2196/18419 (PMC7381005; doi:10.2196/18419)
Supplement: Multimedia Appendix 2 [file resprot_v9i6e18419_app2.pdf]

Australian Government
National Health and Medical Research Council
NHMRC

Doctor Darren Gray
Griffith University
School of Population Health
Herston Rd
Herston QLD Australia 4006

Dear Doctor Gray

Subject: Applicant Response – Project Grant APP1046901 – A school-based health education package for the prevention of soil-transmitted helminth infections in China and the Philippines

Thank you for applying for a NHMRC Project Grant for funding commencing in 2013. You are now invited to respond to assessor comments on the above application assessments by submitting your Applicant Response (Rebuttal).

Your Rebuttal must be uploaded into RGMS by **17:00 hrs AEST, Friday, 29 Jun 2012** to ensure that it is provided to the relevant Grant Review Panel (GRP) for consideration along with your application.

Please ensure your Rebuttal is a single PDF document that does not exceed the two page limit (three pages for applications being assessed by the Indigenous Health GRP). To ensure successful submission, the file cannot exceed 2Mb in size. Other formatting requirements and guidance on how to prepare and submit your Rebuttal are provided at **Attachment A**.

As your response is limited in page length, please use your judgement in responding to assessors' comments and address the most important issues. Rebuttals that do not adhere to the requirements outlined in the attachment may be excluded.

Instructions for submitting your Rebuttal are at **Attachment B**. A report of all assessor comments is at **Attachment C**. If you have questions regarding the Rebuttal process, please contact NHMRC's Research Help Centre at help@nhmrc.gov.au or on 1800 500 983.

Yours sincerely

Project Grants Section
NHMRC

19/06/2012

Encl.
A: Applicant Response formatting requirements
B: Instructions for submitting Applicant Responses in RGMS
C: Project Grants Scheme 2012: Assessment report

Australian Government
National Health and Medical Research Council
NHMRC

Attachment A: Applicant Response formatting requirements

**Format:**	A single document converted into a PDF file that **must not** exceed **2Mb** in size. Applicants and RAOs are advised to retain a copy of the response, including a copy of the PDF file they submit.
**Page Limit:**	**Not more than 2 pages.** References and updates to the Chief Investigator's publication list must be included within the page limit.<br><br>Note: If the application is going to be assessed by the Indigenous Health Research Grant Review Panel (IGRP), applicants will be advised in a separate message. If this is the case, applicants will be permitted to use **an additional third page.** This additional page enables applicants to respond to *The Criteria for Health and Medical Research of Indigenous Australians* (refer: http://www.nhmrc.gov.au/_files_nhmrc/file/grants/indighth.pdf).
**Paper Size:**	Standard A4 (210 x 297mm).
**Title:**	Response should be titled "Applicant Response" in centred font with the Application ID in the top right hand corner.
**Margins:**	All margins must be at least 2.0cm.
**Font:**	At least 12 point and Times New Roman only.
**Line spacing:**	Must be set to single or greater.
**Character spacing:**	Spacing must be set to normal. Scale must be set to 100%.
**Web Links:**	Do not include links to additional information on any website in the Applicant Response, excluding references to published peer review journal articles that are only available online.
**Graphics:**	Graphics (pictures, diagrams etc) may be included in the response. The Applicant Response may be printed and reproduced in black and white and any colour graphics must be visible when reproduced in black and white.
**Tables:**	Tabulated information containing text is not considered to be an image or diagram. Text within tables must comply with the above requirements concerning fonts and spacing.
**Labelling Graphs and Images:**	Axes of graphs and labels of parts of images may be in a reduced font. However, the description and/or legends of all graphs and images must comply with the above formatting requirements.
**File name:**	The PDF file must be named in the following format: "ApplicationID - CIA Surname - Applicant Response.pdf" (for example: APP9011023 - Smith - Applicant Response.pdf).

Attachment B: Instructions for submitting Applicant Responses in RGMS

1. Log into RGMS (www.rgms.nhmrc.gov.au)
2. From the left hand menu select '*Results and Rebuttals*'
3. Locate the required application using the relevant filter fields.
4. Click on the properties icon for the application you wish to view.
5. Select the '*Rebuttal*' tab
6. In the left hand sub-menu click on '*Rebuttal*'
7. Use the 'Browse' button to locate your rebuttal file
8. From the drop-down list select '*Yes, Submit my response to NHMRC*'
9. Click 'Save'

Once your Rebuttal has been successfully uploaded into RGMS, you (the CIA) will receive an automated confirmation e-mail from RGMS.

Australian Government
National Health and Medical Research Council
N H M R C

Attachment C: Project Grants Scheme 2012: Assessment report

**Application ID**	APP1046901
**CIA Name**	Doctor Darren Gray
**Application Title**	A school-based health education package for the prevention of soil-transmitted helminth infections in China and the Philippines

Assessor 1

01. Scientific Quality (This includes the clarity of the hypotheses or research objectives, the strengths and weaknesses of the research plan and the experimental design, and the feasibility of the proposed research. For further detail please refer to section 10.1 of the Project Grants Funding Rules for funding commencing in 2013.)

The major strengths of the proposal are the randomised controlled study design, the background of a recently conducted similar trial by the research group in China, the strong collaborative Australia – China and Australia – Philippines research links, and the inclusion of a cost-effectiveness component. A cluster randomised controlled design (9-10 year schoolchildren in 20 intervention and 20 control schools), with two year follow-up and primary endpoints of incidence of soil-transmitted helminth (STH) infection and STH knowledge is appropriate to evaluate the video-based health education package. The successful demonstration of a 50% reduction in STH incidence within Hunan province with the same education package provides strong evidence for the intervention. However, further justification should be provided for the need to demonstrate efficacy in the high prevalence setting of Yunnan province, given available evidence of efficacy in a moderate prevalence setting (Hunan province). The evaluation of such an intervention across a wide range of prevalence and ethnicity-based settings would be required to provide complete generalisability, but would not be a feasible public health research undertaking. Further justification is required for the combination of two trials in different countries within the one proposal, given the considerable additional expense and duration for completion of both trials. Again, in relation to generalisability across South-East and North Asia, areas of endemic STH, evaluation of the same intervention in many countries would not seem feasible.

Assessor 1

02. Significance and/or Innovation (This includes the potential to increase knowledge about human health, disease diagnoses, or biology of agents that affect human health, or the application of new ideas, procedures, technologies, programs or health policy settings to important topics that will impact on human health. For further detail please refer to section 10.1 of the Project Grants Funding Rules for funding commencing in 2013.)

STH infections are endemic in many resource-limited settings, particularly in Asia. They lead to considerable morbidity, particularly among children. Current control strategies, which largely rely on mass drug administration are not adequate in prevention of reinfection. The use of a video-based health education package, although not highly innovative, is very appropriate given the relative simplicity of the intervention and potential incorporation in

school-based health education. The intervention would appear to have considerable potential public health impact.

Assessor 1

03. Track Record - relative to opportunity (Track record is considered in relation to opportunity and in terms of whether an application demonstrates that the investigator(s) is capable of achieving the proposed project and/or ability to deliver the proposed project in terms of having the appropriate mix of research skills and experience. Where an application involves a CI team, the track record of all CIs is considered. For further detail please refer to section 10.1 of the Project Grants Funding Rules for funding commencing in 2013.)

The proposal has an outstanding CI research team, with considerable experience and international leadership in the infectious disease (particularly neglected tropical diseases) control in resource-limited settings. The prior research undertaken with collaborators in China and Philippines is a major strength of the team, as well as the breadth of experience across epidemiology, biostatistics, qualitative research, public health, and health economics. Given the short period since PhD award, CIA has an excellent emerging research track record, particularly in relation to peer-reviewed grants. CIB has an outstanding public health research track record. CIC has a very good infectious diseases public health research track record, which should be boosted by his excellent track record in peer-reviewed grant awards over the last five years. CID, CIE, CIF, and CIG bring further breadth to the research team, and all have very good emerging track records.

Assessor 1

Budget Comments

The total budget over the five year study period is around $1.8 million, including around $1.0 million in salary and $0.8 million in DRC. Salary costs include PSP4 (50% in years 3-5) for CIA. It is not stated, but this presumably is to provide salary for CIA following the completion of his ARC Early Career Research Award. Given CIA's emerging excellent research track record he would be highly competitive with regard to ongoing ARC research support following completion of his ECR. Thus, this component of the budget should be dependent on lack of such support. A PSP3 (20% for 5 years) is requested for a graduate research assistant to provide data management and programming support. A PSP2 (50%,100%,100%,100%,50%) is requested for a graduate research assistant to undertake laboratory molecular diagnostic work on faecal samples. The 100% fraction for years 2-4 requires further justification. A PSP4 (100% for 5 years) is requested for a postdoctoral researcher to provide overall study coordination. The PSP4 level requires further justification, as this position could be undertaken by a PSP3. A PSP3 postdoctoral health economist (100% for years 4-5) is requested for the cost-effectiveness analyses. Further justification is required for the 100% fraction. DRC include $70,000 for professional animation services to adapt the video to the Philippines. Further justification is required for the one year period required for this component. The workshop/travel budget of $180,000 appears excessive, including the requirement for seven people from Australia to be involved, and therefore should be further justified. The contribution of governments in China ($225,000) and Philippines ($210,000) for provision of core research staff that has considerably reduced the potential RDC is noted.

Assessor 1

Overall Comments

1. Can further justification be provided for the need to demonstrate efficacy in the high prevalence setting of Yunnan province, given available evidence of efficacy in a moderate prevalence setting?

2. Will the vide-based educational package be incorporated into STH control measures in all moderate – high prevalence settings in China, if shown to be effective in Yunnan, or will further trials be required in other provinces and among other ethnic groups?

3. Can further justification be provided for the incorporation of two trials in different countries within the one proposal?

Assessor 2

01. Scientific Quality (This includes the clarity of the hypotheses or research objectives, the strengths and weaknesses of the research plan and the experimental design, and the feasibility of the proposed research. For further detail please refer to section 10.1 of the Project Grants Funding Rules for funding commencing in 2013.)

The current proposal describes the development and application of an educational video to limit the transmission and impact of soil transmitted helminths in school aged children in highly endemic regions of the world. As pointed out by the investigators, the STHs are among the world's most serious and damaging parasites and have particularly high impact in young children. Targeting educational videos at this demographic in endemic regions is clearly appropriate. The methods proposed by the investigators are sound, well considered and have been published by the investigators in the peer-reviewed literature in previous studies. Thus, the approach is well established. A major strength of the current proposal is its multi-disciplinary approach. Often the term 'multi-disciplinary' is over-used in grant proposals, however, in this instance, this is clearly justified. The proposal will draw on the expertise of parasitologists, epidemiologists, clinicians, educators, an anthropologist, and even a team of animators. This is a distinct advantage and suggests a) the the investigators have clearly considered all aspects of the project and b) that this approach will have a high chance of success. It is one thing to make an educational video, it is another thing entirely to make a video that will resonate with children from diverse cultural backgrounds and achieve a lasting change. The investigators have clearly considered this aspect in their project design.

My one query with the investigators approach relates to their description of the estimation of cost-effectiveness. It is not clear to me the approach the investigators will take. Perhaps they could provide more detail. Specifically, I presume by GDP the investigators refer to per capite GDP rather than total GDP? More detail here would be useful in my opinion.

Assessor 2

02. Significance and/or Innovation (This includes the potential to increase knowledge about human health, disease diagnoses, or biology of agents that affect human health, or the application of new ideas, procedures, technologies, programs or health policy settings to important topics that will impact on human health. For further detail please refer to section 10.1 of the Project Grants Funding Rules for funding commencing in 2013.)

STHs are among the world's most prevalent, damaging and, to date, neglected parasites. Their impact on young children is well documented in the literature. In recent years, there has been a major global push to drastically reduce the burden of these parasites in endemic regions through mass drug administration programs in schools. However, as the investigators discuss the challenges associated with this approach is that a) drug treatment offers no protection against re-infection, b) will eventually lead to drug resistance and c) in the face of this

resistance, we currently have few alternative efficacious drugs. The drugs are a core component of effective control, but they must also be supported by improved diagnostic tools, improved infrastructure and educational programs in endemic regions. Thus, the proposal here to develop an educational video to supplement MDA programs directly targeted at children is absolutely required for lasting control and, if it is able to achieve the reductions in regions with high endemicity as it has achieved in the mid-range regions the investigators have assessed to date, will indeed by highly significant.
In terms of innovation, on the surface, the authors are proposing an educational video to inform school aged children and provide them with advice to reduce the transmission of STHs. Clearly this is a good idea but not a new one. Where the proposal is innovative is in the approach used to develop the video. By specifically linking with educators, anthropologists and professional animators, the investigators seek to develop an educational video that is adaptable to a range of cultures and specifically customized to resonate with kids in each of the endemic regions under study. The approach is as important as the content.

Assessor 2
03. Track Record - relative to opportunity (Track record is considered in relation to opportunity and in terms of whether an application demonstrates that the investigator(s) is capable of achieving the proposed project and/or ability to deliver the proposed project in terms of having the appropriate mix of research skills and experience. Where an application involves a CI team, the track record of all CIs is considered. For further detail please refer to section 10.1 of the Project Grants Funding Rules for funding commencing in 2013.)
The proposed team is a solid blend of early career researchers and more experienced scientists with a complimentary expertise and excellent track records to date in terms of a) publication numbers, b) citations and journal rankings and c) the ability to attract competitive research grants. Assessment of the publications of the team to date demonstrate that they have extensive knowledge in this field. Based on their reports on previous NHMRC and/or other funding schemes, their research to date has been highly productive. I see nothing in the present proposal to suggest that this research trajectory would not continue here. Between the expertise of the chief investigators and the links they have established through the associate investigators listed, the team appears to have all of the skills necessary to complete the project and the contacts required to ensure access to the regions proposed for study.

Assessor 2
Budget Comments
The overall budget request is both realistic and modest for a project of this scale. The supplementary support from the Chinese and Filipino governments is substantial (~50% of the field costs in each case) and points to the significant local support for this project.

Assessor 2
Overall Comments
I have no additional comments. Overall my opinion is this is a strong application with a high chance of having a significant outcome in the control of a major group of parasites.

Assessor 3
01. Scientific Quality (This includes the clarity of the hypotheses or research objectives, the strengths and weaknesses of the research plan and the experimental design, and the feasibility of the proposed research. For further detail please refer to section 10.1 of the Project Grants Funding Rules for funding commencing in 2013.)

The research methodology description is very good and clearly articulated in most parts, such as the robust methods around collecting and analysing stool samples, conducting formative research with stakeholders and the children themselves as part of the intervention development in the Philippines. However, little information is provided on the exclusion (ie. only schools with video/ television facility) or inclusion criteria that will be used to identify schools for the sampling frame, how many schools will be approached and included in the sampling frame and so on - a critical piece of information for understanding school characteristics in terms of generalisability and program translation. What is the duration of the classroom discussion session following the 12-min video show? How often will students be exposed to the health education package and classroom discussion? The description implies two points, once at the beginning of the study and again at the 5-month mark. If so, what is the rationale for this frequency and duration of classroom discussion? What specific self-reported measures will be used to assess knowledge, attitude, hygiene behaviour? Will they be established, validated and reliable measures? How will behavioural observations be conducted to again ensure reliability and validity of data obtained? Is the sample size calculation sufficiently powered to detect change in other primary outcomes (knowledge of the worms, their transmission, symptoms, treatment and prevention)? It is not explicitly stated, but did the sample size calculation factor in the possible school clustering effect?

Assessor 3
02. Significance and/or Innovation (This includes the potential to increase knowledge about human health, disease diagnoses, or biology of agents that affect human health, or the application of new ideas, procedures, technologies, programs or health policy settings to important topics that will impact on human health. For further detail please refer to section 10.1 of the Project Grants Funding Rules for funding commencing in 2013.)
This research addresses an issue of major global health importance in a neglected area of infectious disease control – infection related to soil-transmitted helminthes in China and the Philippines. Using a single-blinded cluster RCT design with schools as the unit of randomisation, this application aims to examine the effectiveness of an efficacious video-based health education package (12-mins video, combined with classroom discussions, drawing and essay competitions and a pamphlet containing relevant health messages) delivered in school settings to prevent STH infections in children aged 9-10 years. While video- or television-based communication channels are naturally appealing to children, and have potentials for improving knowledge and motivating behaviour change, its public health utility are limited to schools or communities with access to such technology. This limitation aside, a potentially important outcome from this body of research will be the lessons learned from translating and replicating interventions across different countries and cultures. Interventions with demonstrated effectiveness in one setting may not be effective if implemented in different environments, even if well implemented. In this context, the proposed research has the potential to generate insights into understanding what aspects of an efficacious video-based health education intervention must be modified or reproduced so that similar outcomes can be achieve in different settings, with different populations.

Conducting a cost-effectiveness study alongside the two RCTs is important for informing the translation of the health education package into public health policy and practice, and is a strength of this proposal.

Assessor 3
03. Track Record - relative to opportunity (Track record is considered in relation to opportunity and in terms of whether an application demonstrates that the investigator(s) is

capable of achieving the proposed project and/or ability to deliver the proposed project in terms of having the appropriate mix of research skills and experience. Where an application involves a CI team, the track record of all CIs is considered. For further detail please refer to section 10.1 of the Project Grants Funding Rules for funding commencing in 2013.)
CIA has a solid strong record of research achievements, nationally and internationally, for an early career researcher. He is supported by an esteemed research team also recognised internationally for their work in infectious disease control. Overall the CIs and AIs combined represent an impressive mix of expertise, each at varying level of career development. This team should be able to implement the study, maintain the scientific integrity of the research throughout the life of the project, and address the study research questions. Notwithstanding the exceptional research track records of the investigators, the team is heavily biased towards infectious disease epidemiology. Only two members, AIs Liping and Tallo bring to the team some health education and medical anthropology experience. One would hope that their contributions would increase substantially in the course of the research given that the key intervention strategy is the development and evaluation of a health-education video package, and therefore potentially address some of the major gaps identified in this review (see comments in the Overall Assessment section).

Assessor 3
Budget Comments
The requested budget for this study is substantial but not unexpected given that the research will cover two countries across five years. The in-kind support that will be provided by the China and the Philippines authorities demonstrate the commitments and collaborative nature of this research. That said, this reviewer is uncertain as to why a salary package is required for CIA for the last three years of the research ($123,826.50); could not the considerable analyses be done under the supervision of CIA by a Professional Research Person (PSP 4), a postdoctoral researcher proposed to be employed 1.FTE for the duration of the study period? Similarly, for the requested Professional Research Person ($139,782) of a postdoctoral health economist could not this position be filled by an experienced research assistant (or a PhD candidate)?

Assessor 3
Overall Comments
The investigators highlighted that this proposal is 'assessing the generalisability of our findings in different geographical areas...will provide evidence base for translation of the package into public health policy and practice...' Yet the methodology outlined is heavily biased towards demonstrating the impact of the health video on preventing STH infection. Without doubt it is important to establish effectiveness. However, equally important for this type of research is taking a strategic translation research frame, which would involve collecting and analysing information to assist the investigators to better understand generalisability issues relevant to translating a potentially effective health education package from China to a very different setting and population such as the Philippines. Such data includes collecting/ analysing information around differential effectiveness across schools, factors influencing intervention implementation within schools in relation to effectiveness (intervention fidelity or implementation adherence by schools/teachers), students' experience with the intervention – all relevant for informing in part the development and refinement of the education package in the Philippines, as well as the overall research methodology. The apparent lack of data collection in this regard is a deficiency in this proposal, as it fails to maximize on the generalisability/ replication focus of this body of work. Finally, the video-based health education package is obviously a critical component of this study. The formative

research in the Philippines would focus on adapting the 'The Magic Glasses' to suit the Philippines culture and contexts. As such, please comment/ elaborate on how the research team would address the issue of children's preferences for other health communication strategies such as live dramas, theatres, radios etc should and if this matter arises during formative research?

Indigenous criteria comments (if applicable)
